# An integrated view of metabolic and physiological regulation of toxin production in *Clostridioides difficile*

**DOI:** 10.1128/jb.00318-26

**Published:** 2026-09-14

**Authors:** Chetna Dureja, Ann M. McKelvey, Julian G. Hurdle

**Affiliations:** 1 Center for Infectious and Inflammatory Diseases, Texas A&M Institute of Biosciences and Technology 26514 , Houston, Texas, USA; 2 Department of Translational Medical Sciences, Naresh K. Vashisht College of Medicine, Texas A&M Health Science Center, Houston, Texas, USA; Indian Institute of Technology Bombay, Mumbai, Maharashtra, India

**Keywords:** virulence, pathogenesis, toxin production, sporulation

## Abstract

*Clostridioides difficile* is a leading cause of antibiotic-associated diarrhea, with pathogenesis driven primarily by the glucosylating toxins TcdA and TcdB. Traditionally, toxin gene expression has been explained through regulation of the pathogenicity locus by global transcriptional regulators that integrate nutrient availability, growth phase, and environmental signals. While this framework provides a foundation for understanding toxin regulation, recent advances have broadened this perspective to include diverse metabolic and physiological inputs, including carbon and amino acid metabolism, metal ion homeostasis, redox balance, and cellular energy status. Collectively, these factors suggest that toxin gene expression is a dynamic emergent property that reflects a high level of integration of metabolic and physiological networks. In this minireview, we discuss canonical mechanisms of toxin regulation alongside new insights that highlight population heterogeneity, energy demand conflicts, metabolic constraints, purine metabolism, and cross-regulatory networks linking sporulation and virulence. We further examine discoveries from small-molecule chemical genetics demonstrating that perturbation of metabolic pathways can suppress toxin biosynthesis without substantially affecting bacterial viability. Together, these findings redefine toxin regulation as a systems-level process in which metabolic and physiological states mediate toxin production and contribute to phenotypic heterogeneity. Elucidating these networks may reveal promising targets for the development of anti-virulence strategies for *C. difficile* infection.

## INTRODUCTION

*Clostridioides difficile* infection (CDI) is a diarrheal disease that commonly occurs following disruption of the intestinal microbiota, particularly after antibiotic exposure in elderly patients. In the United States, CDI causes an estimated 500,000 infections and nearly 30,000 deaths annually (1). Although most antibiotic classes are risk factors for gut dysbiosis and CDI development, β-lactams and fluoroquinolones pose the greatest risk by profoundly eroding colonization resistance. Loss of key commensal taxa, including Bacteroidetes and Firmicutes, reshapes the intestinal metabolic landscape, leading to the accumulation of primary bile acids and increased availability of amino acids and other nutrients that promote spore germination and *C. difficile* growth, respectively (2, 3). These changes reshape the intestinal metabolic landscape, providing nutrients and signals that influence *C. difficile* toxin production and virulence. Thus, the CDI environment reflects a metabolic opportunity for *C. difficile* to exploit a depleted microbiota niche.

CDI disease is primarily mediated by two large glucosyltransferase toxins, TcdA (~308 kDa) and TcdB (~270 kDa). By glucosylating and inactivating Rho-family GTPases, they disrupt actin cytoskeleton organization, thereby compromising epithelial barrier functions, triggering inflammation, and shifting the metabolic milieu of the gut in a manner that favors *C. difficile* growth and virulence (4–9). Some epidemic lineages encode a third toxin, the *C. difficile* binary toxin, that is linked to enhanced colonization, epithelial damage, and increased disease severity (10, 11). However, strains lacking TcdA and TcdB are avirulent (7, 8, 12). Expression of the genes encoding the TcdA and TcdB toxins is tightly controlled at the pathogenicity locus (PaLoc) via an intricate network that integrates local transcriptional control with metabolic cues, including carbon and amino acid availability, and redox balance (13–18). Several studies have expanded this view, showing that the regulation of toxin biosynthesis is embedded within the cell’s broader physiological network and intersects with sporulation, metal homeostasis, secondary messenger signaling, and the gut’s nutrient landscape (6, 19–22). While sporulation and toxin production are critical virulence determinants, they are both metabolically demanding processes that respond to nutrient limitation and other environmental stresses. Accordingly, *C. difficile* populations exhibit heterogeneity in toxin production and sporulation, with subpopulations expressing toxin genes, sporulation genes, or both programs (19, 23). We propose that toxin production is not governed by a single regulatory pathway but instead reflects the integration of multiple metabolic and physiological inputs through interconnected regulatory networks. These states are coordinated through both established and yet-to-be-defined regulatory and biochemical pathways that integrate nutrient availability with cellular metabolism. Defining this network and understanding how metabolic perturbations or physiological constraints suppress toxin production may provide new opportunities to develop non-antibiotic anti-virulence strategies for CDI. In this minireview, we examine classical and emerging findings that position metabolism as a central driver of *C. difficile* toxin production, focusing specifically on pathways influencing toxin biosynthesis. We note that broader aspects of *C. difficile* metabolism have been comprehensively reviewed elsewhere (24, 25).

### The pathogenicity locus is a central regulatory node

TcdA and TcdB are chromosomally encoded within a 19.6-kb PaLoc, which also harbors key regulatory and secretory components (26, 27). Central to this locus is the alternative sigma factor TcdR, which is the primary activator of *tcdA* and *tcdB* transcription. TcdR-dependent transcription has been consistently supported by genetic, biochemical, and transcriptional analyses, establishing it as the dominant positive regulator of *tcdA* and *tcdB* transcription (28, 29). TcdR also autoregulates its own expression by recruiting RNA polymerase to its upstream promoter region, thus creating a positive-feedback loop that regulates PaLoc transcription (30). In contrast, TcdC, the putative anti-sigma factor, is proposed to function as an antagonist of TcdR activity, although its precise molecular function remains unresolved (31). While *in vitro* studies suggest that TcdC interferes with the formation of the TcdR–RNA polymerase holoenzyme, potentially through DNA binding, multiple independent analyses have challenged this model, leaving its precise role unresolved (31, 32). Notably, epidemic ribotype 027 strains harbor a frameshift mutation in *tcdC* that was historically linked to increased cytotoxicity (33, 34), although a direct mechanistic relationship between *tcdC* frameshift and toxin hyperproduction remains debatable. The PaLoc also encodes TcdE, a holin-like protein involved in toxin release through pore formation in the cytoplasmic membrane, and TcdL, an endolysin-like protein proposed to facilitate TcdB translocation through these pores (35–37). Transcriptional analysis indicates that the PaLoc is polycistronic (38), with *tcdA*, *tcdB*, *tcdR*, and *tcdE* being co-transcribed, while *tcdC* is independently transcribed (39).

PaLoc-encoded regulation represents only one tier of a broader, multi-layered network controlling toxin production (13–17). Multiple global regulators, including CcpA, CodY, and Spo0A, modulate TcdR activity and thereby govern *tcdA* and *tcdB* expression in response to metabolic signals (14, 40, 41). Toxin regulation extends beyond classical transcriptional control and reflects the integration of multiple regulatory pathways within the broader physiological state of cells in both *in vitro* and *in vivo* environments. This is exemplified by perturbations in metal homeostasis, particularly iron and zinc, which have emerged as determinants of toxin production and virulence (20, 42, 43), likely through effects on metal-dependent enzymes and core metabolic processes rather than directly affecting PaLoc regulation (44, 45). Thus, the PaLoc is not an isolated virulence module but represents the regulatory endpoint of a network integrating metabolic state, environmental signals, and global transcriptional control. Below, we examine transcriptional and non-transcriptional mechanisms that underpin this integration.

## TRANSCRIPTIONAL CONTROL OF TOXIN PRODUCTION IN THE METABOLIC CONTEXT

**Fig 1:**
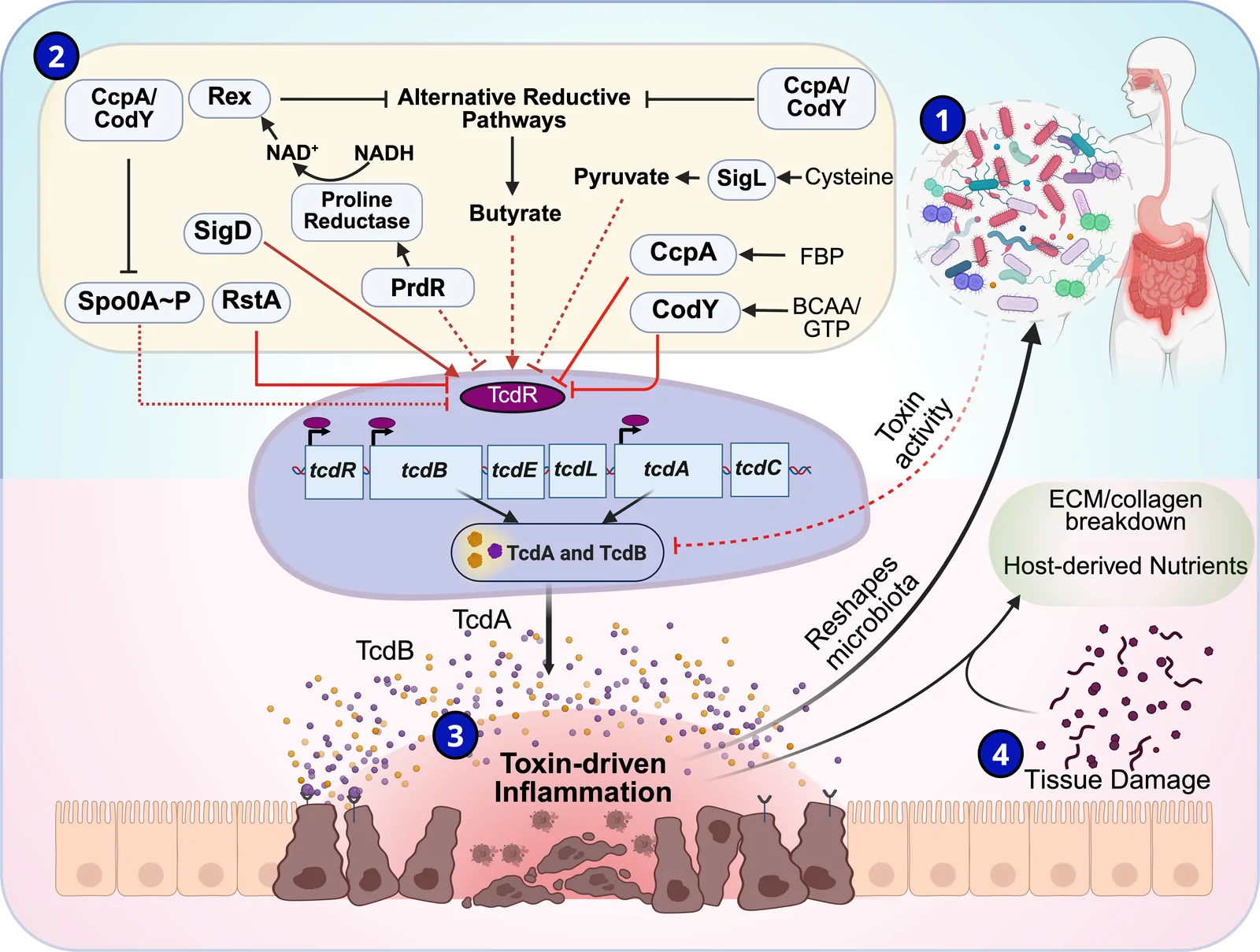
Integrated view of regulation of the *C. difficile* pathogenicity locus (PaLoc) by metabolic and host factors. The PaLoc is comprised of *tcdA*, *tcdB*, *tcdR*, *tcdE*, *tcdC*, and *tcdL*, with TcdR being a key alternative sigma factor. PaLoc is a central node integrating microbiota-, metabolite-, or host-derived signals to control toxin production. (1) The microbiota helps shape the intestinal metabolite landscape through nutrient competition and metabolic activity that influences toxin production. (2) Intracellular regulators (e.g., CcpA, CodY, PrdR/Rex, SigD, Spo0A ~ P, and RstA) integrate metabolic and physiological cues to modulate PaLoc expression either directly through TcdR-dependent transcription or indirectly through metabolic and physiological pathways. Solid red lines indicate experimentally supported regulatory relationships, whereas dashed red lines indicate indirect or inferred effects. (3 and 4) Toxin production promotes tissue damage and inflammation, leading to reshaping of the structural and physiological gut environment, including host-derived nutrients and microbiota composition, creating a feedback loop that further alters the metabolic environment and influences toxin production.

Transcriptional control of the PaLoc is embedded within a broader regulatory network that couples cellular metabolic state with environmental cues (18). This integration is exemplified by the direct influence of carbon, nucleotide, and amino acid metabolic pathways on transcriptional regulators of toxin production, underscoring how metabolism modulates virulence in *C. difficile* (Fig. 1) (13, 15, 41, 46–50).

### Carbon catabolite repression in toxin regulation

Carbon catabolite repression (CCR) is a central mechanism by which bacteria prioritize favorable carbon sources, such as glucose, while repressing pathways involved in utilizing alternative substrates. In *C. difficile*, CCR is mediated by the catabolite control protein A (CcpA), a LacI/GalR family transcription factor that links carbohydrate uptake via phosphoenolpyruvate:carbohydrate phosphotransferase system (PTS) to global transcriptional control (51). Unlike canonical gram-positive models, CcpA regulation in *C. difficile* is primarily driven by fructose-1,6-bisphosphate, rather than phosphorylation of HPr-Ser45, indicating that glycolytic flux rather than phosphotransfer relay is the dominant determinant of CcpA activity (14). In this context, CcpA links carbon availability and metabolism directly to toxin gene expression. Genome-wide transcriptomics and Chromatin Immunoprecipitation Sequencing indicate that approximately 18% of the *C. difficile* genome responds to glucose availability, with nearly half of these genes directly regulated by CcpA. *C. difficile* CcpA binds to at least 55 *creCD* motifs, controlling ~140 genes spanning carbohydrate transport, fermentation pathways, amino acid metabolism, and early sporulation regulators such as *spo0A* and *sigF*, establishing it as a global metabolic coordinator (13). CcpA-mediated repression of toxin production occurs under carbon-replete conditions, with glucose concentrations ≥0.5% suppressing PaLoc expression. Disruption of CCR, such as through deletion of the PTS enzyme I (*ptsI*), leads to depression of PaLoc expression (14). Mechanistically, CcpA directly binds regulatory regions within the PaLoc, including *tcdA*, *tcdB*, *tcdR*, and *tcdC*. Notably, two *C. difficile*-specific *creCD* sites upstream of the *tcdR* promoter indicate that repression is largely mediated through the control of *tcdR*, positioning CcpA as a metabolic gatekeeper of toxin gene expression (13, 14). Beyond direct regulation of PaLoc, CcpA functions epistatically with other global regulators, including CodY, Rex, Spo0A, and SigL, thereby integrating carbon, nitrogen, and redox cues into a coordinated regulatory network (13, 15, 41, 52). Through these interactions, CcpA aligns virulence expression with carbohydrate availability and overall cellular metabolic state.

### CodY and nutrient sensing in toxin regulation

CodY complements CcpA activity by functioning as a central regulator of nitrogen and energy status, linking amino acid availability to toxin gene expression. It coordinates metabolism and virulence by sensing intracellular pools of branched-chain amino acids (BCAAs; leucine, isoleucine, and valine) and GTP, which together reflect cellular nutritional status (16). Under nutrient-rich conditions, elevated BCAAs and GTP enhance CodY binding to the *tcdR* promoter region, thereby repressing expression. Gel-shift and DNase footprinting demonstrate that CodY binds multiple sites within the *tcdR* regulatory region, including sequences overlapping predicted sigma-factor binding motifs, and this interaction is strengthened in the presence of its ligands (41). Conversely, during nutrient limitation, reduced amounts of intracellular BCAA and GTP relieve CodY repression, allowing *tcdR* transcription and subsequent toxin production (41). Thus, CodY provides a direct mechanistic link between nutrient availability and virulence gene expression. However, CodY functions in concert with CcpA. Although these regulators bind distinct promoter regions, there is extensive overlap between their regulons, enabling coordinated sensing of carbon and nitrogen metabolism (13). This epistatic connection positions CodY and CcpA as complementary sensors that fine-tune toxin production in response to nutrient fluctuations.

CodY also intersects with cyclic-di-GMP signaling through control of the SigD-TcdR axis (17, 21, 22). Cyclic di-GMP is a second messenger molecule synthesized from GTP by diguanylate cyclases and degraded by phosphodiesterases (PDEs) (53). Under nutrient-rich conditions, CodY represses expression of the PDE PdcA, thus sustaining high intracellular cyclic-di-GMP that suppresses SigD-dependent activation of *tcdR* (22). Conversely, relief of CodY lowers cyclic-di-GMP, enhances SigD activity, and promotes toxin gene expression (21, 22). The master regulator SinR provides a further layer of regulation by functioning as a cell state-switch that influences the activity of CodY (54). As nutrient levels decline, CodY repression of the *sin* locus is relieved, allowing increased SinR activity, which promotes physiological states that are permissive for motility, sporulation, or toxin production. Although SinR does not directly regulate *sigD*, its regulatory activity aligns with SigD-mediated activation of *tcdR* (55). CodY therefore functions as a key metabolic governor of a tightly interconnected network, integrating nutrient availability, second messenger signaling, and global regulatory networks to align toxin production with cellular physiology.

### Stickland fermentation modulates toxin production

Stickland fermentation in *C. difficile* is closely linked to virulence and is a major metabolic pathway in *C. difficile* that couples oxidation of electron donor amino acids to the reduction of electron acceptor amino acids to support ATP production and redox balance (15).

Within the reductive arm of Stickland metabolism in *C. difficile*, glycine and proline serve as terminal electron acceptors, with proline being preferred (15). Proline reduction is mediated by the *prd* operon, whose expression is regulated by the PrdR transcriptional regulator (15, 56). Interestingly, a *prdR* mutant showed a 2.5-fold reduction in *tcdA* expression, even in the presence of proline (15). Additional regulation of the *prd* operon involves the alternative sigma factor SigL (σ54/RpoN). Clark et al. identified a SigL-dependent promoter associated with the *prd* locus and showed that loss of *sigL* reduced the abundance of PrdA, a component of the proline reductase complex, and PrdF, a proline racemase, in two genetic backgrounds, BI-1 (ribotype 027) and CDC1 (ribotype 078) (57). In a separate study, Yang et al. reported that in CD630 (ribotype 012), loss of *sigL* was associated with marked reductions in expression *of prdB* and *prdF*, increased expression of *tcdA* and *tcdB*, and a 100-fold increase in cytotoxicity toward Vero epithelial cells (58). Notably, the *prd* operon is also positively regulated by CcpA, providing a point of integration between carbohydrate and amino acid metabolism (13). Collectively, these findings suggest that Stickland metabolism contributes to toxin regulation by integrating amino acid availability with broader metabolic networks. *C. difficile* also encodes a transcriptional repressor Rex, which has DNA-binding activity that is attenuated by NADH and NADPH. In *S. aureus*, Rex is involved in adapting cells to oxygen stress by derepressing genes involved in nitrate respiration and fermentation (promoting fermentation products such as lactate, formate, acetate, and 2,3-butanediol) (59, 60). In *C. difficile*, the Rex ortholog regulates genes involved in Stickland metabolism and NAD^+^ regeneration. However, although *rex* mutants can exhibit increased toxin levels *in vitro*, reduced virulence in the hamster model of CDI was reported, and most likely reflects broader metabolic effects, indicating that the impact of Rex on toxin production is context dependent (61).

### Metabolism and toxin production during infection

During infection, the activity of toxins remodels the host and microbial environment, driving inflammation, suppressing competing taxa, and liberating host-derived peptides that are exploited by *C. difficile* (4–6, 62). Within this environment, *C. difficile* reprograms its metabolism, characterized by increased expression of carbohydrate uptake systems and downregulation of biosynthetic pathways, including BCAA synthesis, indicating preferential utilization of host-derived nutrients (6, 63). Toxin-mediated degradation of extracellular matrix proteins further releases amino acids, including proline, hydroxyproline, and collagen-derived peptides, which fuel Stickland fermentation to support energy generation and redox balance (64). At the community level, inflammation suppresses recovery of competing taxa, such as Bacteroidaceae, reinforcing a nutrient landscape that favors *C. difficile* (6, 65).

Although much of the mechanistic understanding of CcpA and CodY is derived from *in vitro* studies, recent *in vivo* investigations have clarified how these regulators coordinate metabolism during infection. Notably, naturally occurring CodY variants with single amino acid substitutions in the GTP- or BCAA-binding pockets exhibit reduced binding to the *tcdR* promoter, resulting in increased toxin production and enhanced virulence in hamsters (66). Variants such as Tyr146Asn and Val58Ala, which impair GTP and BCAA binding, respectively, recapitulate this phenotype in isogenic strains, directly linking CodY ligand sensing to virulence *in vivo* (66). Given the role of CodY during infection, it is not surprising that BCAA and butyrate biosynthesis are downregulated, indicating a shift toward using host-derived nutrients rather than *de novo* synthesis. Systems-level analyses, using EGRIN and PRIME, further demonstrate that CodY and CcpA coregulate pathways involved in carbohydrate transport, Stickland fermentation, nucleotide metabolism, and PaLoc expression (67). Disruption of both regulators results in growth defects and reduced virulence *in vivo*, reflecting diminished fitness within the gut ecosystem. Consistent with this, *C. difficile* CcpA/CodY mutants are outcompeted by *Paraclostridium bifermentans*, which, like *C. difficile*, also adopts Stickland metabolism (65, 67). Metabolomics on CDI patient stools have identified Stickland fermentation signatures and end-products suggesting that this pathway is active during infection (68, 69). A further example is evident from co-infections of *C. difficile* and *Enterococcus faecalis*, which is a risk factor for severe CDI in humans. It was shown that *E. faecalis* influences the gut metabolic environment in CDI by depleting arginine while producing ornithine and leucine that are utilized by *C. difficile* for Stickland fermentation, enhancing its growth and toxin production (70). However, this interaction may not be universal, as some *E. faecalis* strains can suppress *C. difficile* (71), highlighting that variations among species may influence interaction outcomes in the gut.

### Cross circuit control of toxin production and sporulation

**Fig 2:**
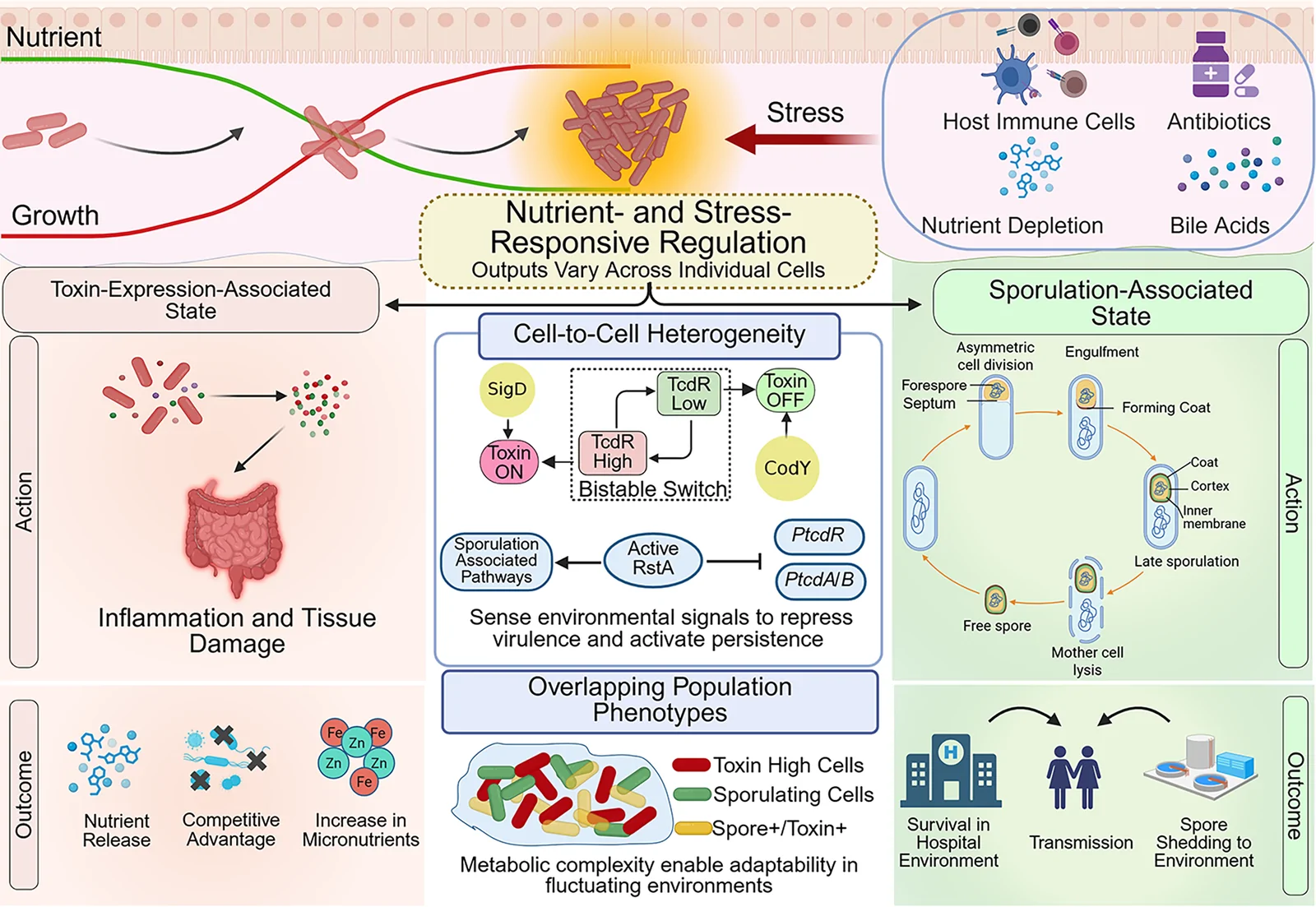
Population heterogeneity in toxin expression and sporulation. Following successful colonization, *C. difficile* appears to encounter nutrient limitation and host-associated stresses that activate nutrient- and stress-responsive regulatory pathways. These inputs contribute to cell-to-cell heterogeneity in toxin expression and sporulation-associated programs through regulators including TcdR, CodY, SigD, RstA, and Spo0A. TcdR-dependent bistability contributes to toxin ON and OFF states, while RstA represses toxin-associated transcription and influences sporulation-associated pathways in a strain-dependent manner. These regulatory networks generate overlapping population phenotypes, including toxin-high cells, sporulating cells, mixed cells, and cells with low or undetectable reporter activity, indicating that toxin expression and sporulation are condition-dependent and can overlap in individual cells.

During the stationary phase, in nutrient-limited settings, *C. difficile* confronts a critical resource allocation problem, as both toxin production and sporulation are energetically costly processes (Fig. 2). Single-cell analyses indicate substantial heterogeneity in both toxin production and sporulation (19). This heterogeneity is consistent with a bet-hedging model that enables populations to balance survival and transmission, with toxin production contributing to host damage and sporulation facilitating persistence and dissemination. However, other evidence suggests that there is not strict segregation between these outcomes, and the amount of overlap between these physiological states appears dependent on environmental conditions and genetic background (19, 23).

Using reporter strains expressing the gene encoding mCherry from *tcdA* promoter, Weiss and colleagues showed that at the single-cell level, toxin gene expression is bimodal, with only a subset of stationary-phase cells entering a toxin-ON state. This was governed by TcdR acting as a bistable switch due to cell-to-cell variations in the accumulation of TcdR in cells. This bistable behavior is further modulated by global regulators, including SigD, which biases cells toward toxin expression, and CodY, which promotes a toxin-OFF state in cells with sufficient nutrients. However, neither SigD nor CodY was required for bimodal toxin expression, which both highlights the complexity and contribution of additional regulatory inputs (23) (Fig. 2).

Extending these findings, Shen and colleagues used dual-reporter analyses to simultaneously monitor toxin gene expression and sporulation at the single-cell level (19). They revealed that toxin production and sporulation are both heterogeneous and environmentally dependent. For example, cultures grown in tryptone yeast broth contained toxin-ON and sporulation-ON subpopulations, including a subset of cells positive for both reporters. In contrast, cells from cultures grown on agar media exhibited reduced overlap in signals for toxin and sporulation reporters, suggesting that environmental conditions may influence the extent to which these processes coexist within individual cells. These data support a model in which toxin production and sporulation are coordinated and condition-dependent processes rather than universally segregated cell fates (19). Indeed, toxin expression has been observed in sporulating cells, suggesting that they are not mutually exclusive programs (19, 23, 72). In mice infected with *C. difficile*, single-cell analysis of reporter strains also revealed that heterogeneity in toxin expression occurs *in vivo* but was not spatially restricted to regions of the gut (Fig. 2) (73).

Subpopulations exhibiting differences in toxin production, colony morphology on agar, motility, and pathogenicity have been further characterized in studies of phase variation by Tamayo and colleagues (74–76). *C. difficile* naturally undergoes phase variation, in which DNA inversions, including the flagellar, *cmr*, and *pdcB* switches, generate distinct cell states with altered motility, morphology, c-di-GMP signaling, and sporulation behavior (74–77). Among these, flagellar switching has a particularly strong influence on toxin production. It involves inversion of a 154-bp segment upstream of the *flgB* operon, which modulates the expression of the operon, including *sigD*; the 154-bp invertible element contains regulatory sequences that control operon expression by influencing transcriptional elongation and termination (76). Because SigD positively regulates *tcdR*, flagellar switching indirectly controls *tcdA* and *tcdB* expression by attenuating SigD-TcdR interaction. Cells in the flg-ON state are motile and exhibit increased toxin production, whereas flg-OFF cells are nonmotile, display reduced *sigD* expression, and show attenuated toxin production and virulence in animal models. Noteworthy, flg-OFF can become flg-ON again, which begs the question as to its benefit. Flg-OFF cells may be less metabolically active, appearing as smooth colonies on agars and are better at avoiding immune clearance compared to flg-ON cells (76). Thus, switching between flg-OFF and flg-ON phenotypes via invertible DNA recombination provides *C. difficile* with a flexible and adaptable population structure, enabling it to be dynamic in its response to changes in environmental challenges, e.g., nutrient availability and host immune pressures. Recent studies further show that increased heterogeneity in flagellar gene expression, driven by variation in the flagellar switch region, promotes virulence in ribotype 027 strains (78).

## INHIBITION OF TOXIN PRODUCTION BY SMALL MOLECULES AND METABOLIC REPROGRAMMING

While the above studies firmly establish the complexity of *C. difficile* pathobiology, an intriguing question is whether these interconnected networks can be exploited to attenuate *C. difficile* pathogenesis. Chemical genetic studies involving small molecules have shown that perturbation of metabolic and physiological processes can suppress toxin production and provide new insights into mechanisms that underpin virulence (79–81). Although some compounds may exert pleiotropic effects, they have proven valuable for identifying druggable metabolic and physiological pathways that can abrogate toxin production. Accordingly, this section of the minireview focuses on insights from small-molecule chemical genetics, microbial genetics, and systems-level analyses demonstrating that perturbations of metal homeostasis (iron and zinc), membrane energetics, central carbon and nucleotide metabolism, and intracellular redox balance are associated with reduced toxin biosynthesis. Exemplary studies indicating suppression of toxin production by small molecules include ebselen (redox and thiol stress), secondary bile acids (affects vegetative growth), enoxolone (purine salvage, ATP, and phosphate metabolism), urolithin A (mechanism unresolved), and meclizine (carbohydrate metabolism) (3, 79, 81–83). These mechanisms are discussed below.

### Role of metal ion homeostasis in *C. difficile* pathogenesis

**Fig 3:**
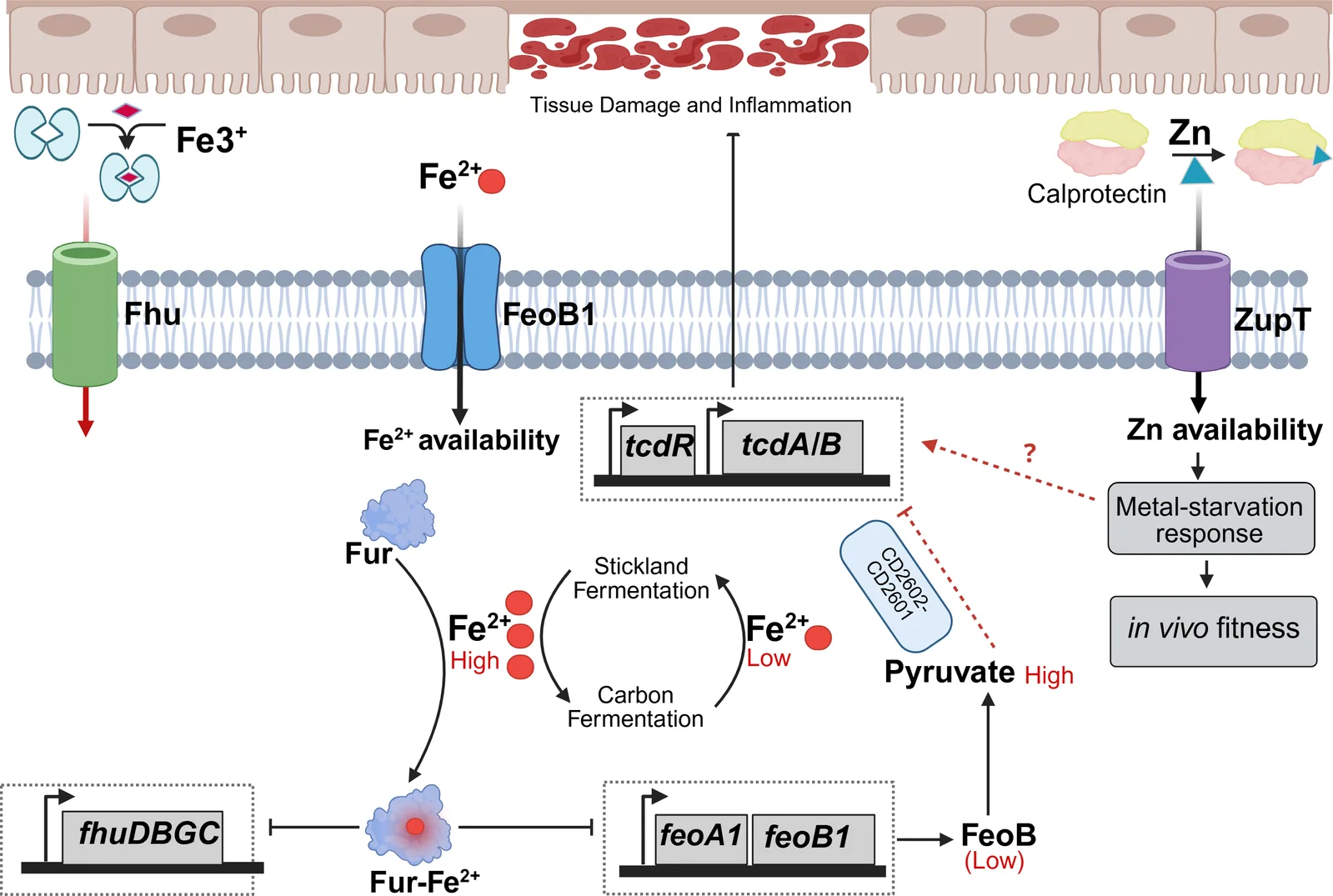
Metal-ion homeostasis intersects with metabolic pathways linked to toxin production in *C. difficile*. Iron and zinc availability influence metal-dependent cellular processes, including central metabolism, redox balance, and adaptation to host-imposed nutrient limitation. Transporters FeoB1, Fhu, and ZupT are involved in metal acquisition. Reduced FeoB1-dependent ferrous iron uptake limits intracellular iron, disrupts iron-dependent metabolism, and is associated with pyruvate accumulation, reduced *tcdA/tcdB* expression, and decreased toxin production (43). Elevated pyruvate has been linked to repression of the *CD2602-CD2601* two-component system, although the mechanism connecting this pathway to toxin reduction is unresolved (52). Zinc availability is constrained by calprotectin and depends in part on ZupT-mediated uptake, which affects metabolic adaptation and *in vivo* fitness (84, 90). Direct effects of zinc limitation on toxin gene expression or toxin abundance remain to be established. Solid arrow indicates an experimentally supported relationship, whereas dashed arrow indicates indirect or inferred links.

Metal ion homeostasis represents an important layer of metabolic regulation that influences *C. difficile* physiology, fitness, and pathogenesis (Fig. 3). Although direct metal-dependent regulation of toxin production remains incompletely defined, perturbations in metal availability are reported to alter metabolic and physiological states that influence toxin production and pathogenesis (43, 84). While this area is in its infancy in *C. difficile*, the acquisition and utilization of essential metals are well characterized in other bacterial pathogens. In organisms such as *Staphylococcus aureus*, *Salmonella spp*., and *Pseudomonas aeruginosa*, metal ions including iron, zinc, and manganese are vital for oxidative stress resistance, DNA synthesis, and regulation of virulence (85, 86). These pathogens employ diverse metal uptake systems, including siderophores, zinc transporters, and manganese acquisition pathways, to survive in metal-limited host environments (87). Importantly, metal acquisition also influences different metabolic pathways linked to pathogenicity, including toxin production, biofilm formation, and immune evasion (88). In *C. difficile*, emerging evidence indicates that metal homeostasis influences cellular metabolism and virulence. For example, dysregulation of iron and zinc availability perturbs metalloenzyme-dependent cellular reactions, including in central carbon metabolism and associated redox processes, which may indirectly affect toxin production through changes in cellular physiology (Fig. 3) (44, 89).

### Ferrous iron role in *C. difficile* pathogenesis

Iron is an essential micronutrient, which is sequestered by the body in the form of hemoglobin, ferritin, transferrin, calprotectin, lipocalin-2, and lactoferrin. Bacterial iron acquisition is even more complex in the gastrointestinal tract, where interspecies competition for this micronutrient is necessary for their survival. It is thought that within the anaerobic gut, ferrous iron (Fe^2+^, the reduced state of iron) is the most abundant form of iron available to bacteria, while less available ferric iron (Fe^3+^) may also be used by bacteria through siderophore systems (91, 92). Most *C. difficile* strains do not appear to encode siderophores, which are small molecules with high affinity coefficients for Fe^3+^ (e.g., K_f_ 10^30^ to 10^52^ M^−1^). However, *C. difficile* expresses siderophore receptors, such as FhuDBGC, allowing it to capture iron from siderophores and ferrichrome, produced by other organisms (93). Inactivation of FhuDBGC by deleting the siderophore-binding protein FhuD was shown to attenuate *C. difficile* growth in iron-defined medium, which was rescued by providing other iron sources. The FhuD mutant grew on different siderophores, including hydroxamate (e.g., arthrobactin), catecholate (e.g., enterobactin), and phenolate (e.g., pyochelin), indicating that *C. difficile* may have other mechanisms of acquiring iron from siderophores. This view is somewhat supported by the fact that loss of FhuD did not attenuate virulence in a murine model of CDI. However, both *in vitro* and *in vivo* studies support a link between iron availability and toxin production. For example, *tcdA* and *tcdB* transcription has been shown to be elevated in media containing excess iron, while iron chelators suppress their transcription (20). *C. difficile* encodes three ferrous iron transporter systems (FeoB1 to FeoB3), of which FeoB1 is the most upregulated transporter in response to low iron conditions, both *in vitro* and *in vivo* (43, 45). FeoB1 is thought to be the main ferrous transporter in *C. difficile*. Consistent with this, it was reported that deletion of FeoB1 caused an approximately 20% decrease in intracellular iron content. Transcriptomic analysis revealed that deletion of *feoB1* was associated with upregulation of genes involved in iron acquisition (43). Notably, cells upregulated flavodoxin (*fldx*), an iron-independent flavin cofactor that can substitute for ferredoxin, along with genes encoding the Fhu, ferrichrome transporter, FeoB2, and the low-affinity zinc transporter ZupT, which can also transport iron. Consistent with reduced intracellular iron, the mutant also downregulated the iron storage protein bacterioferritin. Deletion of *feoB1* was also associated with intracellular accumulation of pyruvate (~30% increase), with a concomitant decrease in expression of pyruvate carboxylase (Pyc), which converts pyruvate to oxaloacetate (43). Pyruvate is known to suppress toxin production, although the underlying mechanism(s) remains unresolved, but alterations in energy flux through central carbon metabolism or changes in redox balance are likely contributing factors (Fig. 3) (52). Consistent with this, the *feoB1* mutant exhibited marked downregulation of *tcdA* and *tcdB*, resulting in decreased toxin production and reduced cytotoxicity against Vero epithelial cells. In a murine model of CDI, the virulence of the FeoB1 mutant was markedly attenuated, with fecal and cecal contents showing significantly reduced *C. difficile* bioburdens, toxin levels, and decreased expression of inflammatory markers TNF-α, GM-CSF, and IL-10 (43). Collectively, these findings position FeoB1 as an important determinant of iron homeostasis, metabolic adaptation, toxin production, and virulence in *C. difficile*.

Further supporting the importance of iron homeostasis in CDI, loss of ferrous iron storage in ferrosomes, encoded by *fezA* (a membrane protein) and *fezB* (P_1B6_-ATPase transporter), has been shown to affect pathogenesis in mice (94). Ferrosomes function as intracellular reservoirs to buffer iron availability, enabling *C. difficile* to adapt to fluctuating iron conditions by releasing ferrous iron when needed. Consistent with the loss of *feoB1* phenotype, the deletion of *fezB* in R20291 did not affect growth in rich media but resulted in significantly reduced bacterial burden and attenuated disease in mice with CDI, supporting a role for ferrous iron storage in the *in vivo* fitness and pathogenesis of *C. difficile* (94). These findings suggest that disruption of ferrous acquisition and storage can impair *C. difficile* fitness in the gut, although whether ferrosome-dependent iron storage directly influences toxin production remains to be determined.

### Zinc’s role in *C. difficile* pathogenesis

Zinc has also emerged as a pivotal determinant of *C. difficile* fitness during infection, operating at the interface of bacterial physiology and host-imposed nutritional immunity. Unlike many Firmicutes that encode multiple high-affinity ABC-type zinc uptake systems, *C. difficile* depends on the ZIP-family metal transporter ZupT to acquire zinc under metal-limited conditions. Deletion of *zupT* impairs growth in zinc-depleted media and, in murine models, results in reduced colonization and competitive fitness relative to wild-type strains, highlighting the necessity of efficient zinc uptake for successful colonization and infection (84). The role of zinc homeostasis is underscored by the host response during CDI, where neutrophil-derived calprotectin chelates zinc and other transition metals such as iron and manganese to restrict bacterial proliferation. Transcriptomic analyses have revealed that calprotectin exposure triggers a metal-starvation response in *C. difficile*, including marked upregulation of flavodoxin, ZupT, FeoB1, ferrichrome transporter, and other metal acquisition systems (90). These analyses also revealed that calprotectin induced transcription of proline reductase genes and the fermentation of proline. This coordinated response indicates that calprotectin not only impacts metal ion homeostasis but also energy metabolism and physiological adaptation in *C. difficile* (90). However, direct effects of zinc limitation or calprotectin exposure on *tcdA* or *tcdB* transcription, or on TcdA and TcdB protein levels, have not been examined and require further investigation. These findings nevertheless position zinc homeostasis as a determinant of the metabolic and physiological state of *C. difficile*, which may influence toxin production indirectly through nutrient and energy metabolism.

### Disruption of cellular redox control by inhibition of thioredoxin reductases

Intracellular redox homeostasis has also recently emerged as a non-canonical regulator of toxin biosynthesis. This is exemplified by ebselen and auranofin, which are redox-active molecules (80, 95). Ebselen is an organoselenium drug candidate with antioxidant activity by inhibiting thioredoxin reductases (Trx). It has been reported to inhibit *S. aureus* growth by targeting Trx enzymes (96). In the context of *C. difficile*, ebselen was first described as a direct inhibitor of TcdA and TcdB cysteine protease domains, with a lack of antibacterial activity against *C. difficile* in Brucella broth containing blood. Studies by the Hurdle laboratory revealed that ebselen had broader cellular effects in *C. difficile*, depending on media composition. Inclusion of pyruvate and blood in the media reduced the antimicrobial activity of ebselen, as they likely protected against reactive stress. At sub-inhibitory concentrations, ebselen inhibited both toxin production and sporulation. Key physiological signatures from ebselen-treated cells suggested disruption of thiol metabolism and cellular redox, with associated increases in intracellular cysteine, depletion of low-molecular-weight non-protein thiols, and disruption of the NAD+/NADH ratio. Ebselen also induced transcription of genes involved in Stickland metabolism, including proline reductase genes, components of the Rnf complex, and ATP synthase subunits (80). As indicated above, Stickland fermentation allows *C. difficile* to balance reducing equivalents by coupling the oxidation of electron donor amino acids to the reduction of electron acceptor amino acids such as proline. Hence, induction of the *prd* locus by ebselen, together with NAD+/NADH imbalance, might suggest that *C. difficile* compensates for thiol-redox stress by driving Stickland metabolism to regenerate NAD+. Interestingly, overexpression of genes encoding individual Trx system proteins did not rescue cells from ebselen, and strains belonging to ribotype 078, which encode a fourth Trx enzyme, were intrinsically ebselen resistant. Ebselen also downregulated regulatory loci associated with toxin and sporulation networks, including *agrB/agrD* and *spo0A*-associated responses, while reducing toxin abundance and spore formation. Overall, the cellular effects of ebselen are consistent with toxin production and sporulation being highly sensitive to perturbations in thiol homeostasis and NAD+/NADH balance, although the underlying mechanisms remain incompletely resolved (80). This model is partly supported by studies with auranofin, an inhibitor of bacterial thioredoxin reductases, which inhibits *C. difficile* growth, toxin production, and sporulation at concentrations below the MIC (95). However, there is a need for advanced mechanistic studies that define effects of auranofin on *C. difficile* metabolism and redox balancing.

### Dysregulation of purine, ATP, and phosphate metabolism by enoxolone

Inhibition of adenine deaminase, ATP synthase, and phosphate metabolism has also revealed that metabolic disruption of these pathways also hinders toxin regulation. This discovery followed from the systematic identification of enoxolone, as a small molecule that suppresses toxin production without major inhibition of bacterial growth (81). Enoxolone (18β-glycyrrhetinic acid, a licorice-derived triterpenoid) was identified from a phytochemical screen as a low micromolar inhibitor of toxin synthesis. Enoxolone-treated cultures exhibited reduced TcdA and TcdB protein levels, decreased *tcdA*/*tcdB* transcription, and diminished cytopathic activity against epithelial cells, while maintaining bacterial viability, pointing to activity upstream of toxin production rather than direct toxin neutralization. Mechanistic studies using affinity-based proteomics, genetics, and multi-omics revealed two primary targets: adenine deaminase (Ade) and ATP synthase (81). Ade catalyzes the first committed step of the purine salvage pathway, converting adenine to hypoxanthine, which feeds other steps in the pathway. Genetic silencing of *ade* phenocopied suppression of toxin biosynthesis, while biophysical assays (surface plasmon resonance and isothermal colorimetry) confirmed that enoxolone binds to Ade. Enoxolone treatment caused an accumulation of adenosine and depletion of hypoxanthine, while supplementation with downstream purines partially restored toxin production, indicating that flux through purine metabolism contributes to toxin biosynthesis. Restoration of purine metabolism alone, however, was insufficient to fully rescue enoxolone-mediated toxin suppression, pointing to additional metabolic constraints imposed by enoxolone (81).

Inhibition of ATP synthase has also been implicated in toxin regulation, as moderate depletion of cellular ATP (~25%–33%) using enoxolone or the ATP synthase inhibitor Bz-423 at subinhibitory concentrations, or through genetic silencing of *atpA*, robustly suppressed toxin production without affecting growth (81). Enoxolone also induced a Pho-regulon-like response, marked by the disruption of phosphate levels. Supporting this, silencing *atpA* depleted intracellular phosphate levels, whereas *ade* silencing had no effect, further supporting a role for ATP synthase in phosphate regulation. Although phosphate supplementation restored ATP and phosphate levels, full rescue of toxin production required co-supplementation with hypoxanthine, indicating that enoxolone suppressed toxin biosynthesis by imposing metabolic constraints in purine and phosphate metabolism. Collectively, studies with enoxolone suggest that nucleotide salvage, energy generation, and phosphate homeostasis are important determinants of toxin production in *C. difficile*, although the precise regulatory connections remain incompletely understood.

### Dysregulation of carbohydrate metabolism by meclizine

As discussed above, carbon metabolism plays a central role in regulating toxin production. The identification of meclizine, which induces intracellular accumulation of glucose and pyruvate, highlights that disrupting carbon metabolism can suppress toxin biosynthesis. Meclizine, an antihistamine, was identified through a high-throughput screen for inhibitors blocking *tcdA* promoter activity. Meclizine activity was validated across orthogonal assays, demonstrating that it reduced TcdA and TcdB protein levels and repressed *tcdA*/*tcdB* transcription, with EC_50_ values of ~3–5 µM. The transcriptomic profiles of cells showed that meclizine extensively remodeled central carbon metabolism. This response was characterized by upregulation of multiple PTS carbohydrate transporters and the lactate-to-pyruvate confurcation operon, alongside repression of core glycolytic genes *cggR* (central glycolytic genes regulator), *gapB* (glyceraldehyde-3-phosphate dehydrogenase 2), *pgk*, *pfkA* (phosphoglycerate kinase), *pykF* (pyruvate kinase), and ribosomal protein operons. These transcriptional changes represent an unusual metabolic state, characterized by decoupling of enhanced carbohydrate uptake with altered glycolytic flux and downstream utilization of pyruvate. Consistent with this, meclizine induced intracellular accumulation of glucose (up to ~2.7-fold) and pyruvate (up to ~7-fold) (79). Thus, this supports a model in which meclizine imposes an intracellular carbon-replete state that is disfavored for toxin biosynthesis. More broadly, these findings reinforce the principle that toxin production in *C. difficile* is highly dependent on the metabolic context of cells. However, it is important to note limitations of the meclizine study: the precise molecular targets remain undefined, leaving the causal relationship between drug exposure and metabolic reprogramming unresolved at the target and regulatory level. Although the metabolic state induced by meclizine is compatible with carbon catabolite repression, direct involvement of CcpA was not confirmed. Thus, at present, meclizine is best considered as a probe inducing metabolic reconfiguration, rather than a fully mechanistically resolved inhibitor of toxin biosynthesis. Nevertheless, the associated metabolic changes and toxin suppression phenotype further support a link between cellular metabolic state and toxin biosynthesis.

## CONCLUDING PERSPECTIVE

*C. difficile*, by definition, is a difficult pathogen to study. However, over the past two decades, work from laboratories around the world has greatly advanced our understanding of its complex metabolic and virulence circuitry. In our view, the *C. difficile* field should be proud of how far it has come. Now, we can categorize toxin production in *C. difficile* as a systems-level process that emerges from the integration of metabolic state, environmental signals, and regulatory networks. Rather than being governed by discrete pathways, toxin expression reflects an emergent property of cellular physiology constrained by nutrient availability, redox balance, metal homeostasis, and bioenergetic capacity. Thus, *C. difficile* virulence expression is embedded within its core metabolism, and this therefore makes it inherently targetable for drug discovery. Indeed, advances in CDI animal models, clostridial genetics, systems biology, and chemical biology have established a strong foundation for understanding *C. difficile* pathogenesis. Notably, the convergence of central carbon metabolism, Stickland fermentation, and metal-dependent processes with global regulators highlights key metabolic checkpoints that constrain toxin production and might be targetable. Furthermore, since iron and zinc homeostasis have emerged as important determinants of metabolic flux and *in vivo* fitness, they also represent underexplored opportunities for therapeutic intervention. Nonetheless, we believe that the field is at a key transitional stage that is ready for advanced forward genetics, including transposon mutant screens, to provide an unbiased framework that defines genes and pathways required for *C. difficile* fitness, metabolism, sporulation, and toxin production. When integrated with chemical genetics, these strategies enable condition-specific vulnerabilities to be identified, from which to prioritize metabolic drug targets. Studies with compounds such as enoxolone, ebselen, and meclizine further demonstrate that altering metabolic state can suppress toxin production without inhibiting growth, thereby supporting the concept of pharmacological “metabolic disruption” as an anti-virulence strategy. They also reflect that novel biological insights can be rapidly gained through chemical genetics, which, more so than current clostridial genetics, can overcome bottlenecks of studying diverse *C. difficile* lineages.

We believe that advancements made in defining *C. difficile* pathogenic biology can create new opportunities for discovering narrow-spectrum therapeutics. Even where targets have mammalian homologs, structural and regulatory differences can be exploited to achieve pathogen-selective inhibition by taking advantage of artificial intelligence and structural biology. Although key questions remain, including the dynamics of integrating metabolic signals in a complex milieu, *in vivo*, and at the single-cell level, the field is now positioned to move from descriptive models to predictive, mechanistically informed frameworks. Defining how metabolic constraints govern toxin and sporulation and how these interconnected physiological states are coordinated in different environmental contexts will be central to advancing actionable drug targets. We view this as an exciting next frontier, one that builds on decades of progress, which now offers a clearer path toward developing next-generation anti-virulence therapies to disarm *C. difficile*.
